# Psychotherapie auf Distanz in Österreich während COVID‑19. Zusammenfassung der bisher publizierten Ergebnisse von drei Onlinebefragungen

**DOI:** 10.1007/s00729-021-00168-3

**Published:** 2021-04-23

**Authors:** Thomas Probst, Barbara Haid, Wolfgang Schimböck, Peter Stippl, Elke Humer

**Affiliations:** 1 Department für Psychotherapie und Biopsychosoziale Gesundheit, Donau-Universität Krems, Dr.-Karl-Dorrek-Straße 30, 3500 Krems an der Donau, Österreich; 2 Österreichischer Bundesverband für Psychotherapie, Löwengasse 3, 1030 Wien, Österreich; 3 ABILE-Ausbildungsinstitut für Logotherapie und Existenzanalyse, Bahnhofstraße 3, 3390 Melk, Österreich

**Keywords:** COVID‑19, Psychotherapie, Internet, Telefon, COVID‑19, Psychotherapy, Internet, Telephone

## Abstract

Die Donau-Universität Krems untersuchte in Kooperation mit dem Österreichischen Bundesverband für Psychotherapie mit drei Onlinebefragungen Psychotherapie auf Distanz in Österreich während der COVID-19-Pandemie. Dieser Artikel fasst die bisherigen bereits publizierten Ergebnisse dieser Befragungen zusammen. An der ersten Onlinebefragung, welche in den ersten Wochen des ersten coronabedingten Lockdowns in Österreich stattfand, beteiligten sich insgesamt 1547 Psychotherapeut*innen. Die Ergebnisse zeigen, dass Psychotherapeut*innen während des Lockdowns Psychotherapie im persönlichen Kontakt verstärkt durch Psychotherapie auf Distanz (Telefon oder Internet) ersetzten. Ein erhöhtes Stresserleben sowie jobbezogene Ängste waren v. a. bei Psychotherapeut*innen vorhanden, bei denen Psychotherapie die einzige Einnahmequelle darstellte. Die Erfahrungen mit Psychotherapie auf Distanz wurden als insgesamt positiver beschrieben als sie erwartet wurden. Nichtsdestotrotz wurde Psychotherapie auf Distanz als nicht vollständig vergleichbar mit Psychotherapie im persönlichen Kontakt angesehen. An einer zweiten Onlinebefragung nach dem ersten Lockdown (Sommer 2020) beteiligten sich 222 Psychotherapeut*innen aus Österreich. Ziel dieser Befragung war es den Wechsel des Behandlungsformats (persönlich zu digital oder digital zu persönlich) im Hinblick auf die Anwendung spezifischer therapeutischer Interventionen genauer zu untersuchen. Unter digital wurden verschiede Medien wie z. B. Sprachtelefonie, Videokonferenz, Chats und E‑Mail subsumiert. Zeitgleich mit der zweiten Befragung wurde eine dritte Onlinebefragung durchgeführt, an der 139 Patient*innen der 222 österreichischen Psychotherapeut*innen teilnahmen, um auch die Patient*innen-Perspektive beim Wechsel des Behandlungsformats zu untersuchen. Erste Ergebnisse zeigen, dass Psychotherapeut*innen und Patient*innen beim Wechsel des Behandlungsformats einen Unterschied hinsichtlich der angewandten therapeutischen Interventionen erlebten. So wurden die untersuchten therapeutischen Interventionen als typischer für die Therapie im direkten persönlichen Kontakt als für die Psychotherapie auf Distanz bewertet. Zudem veränderte sich die subjektive Bedeutung verschiedener Bereiche des Lebens während der Corona-Pandemie. Die bisherigen Auswertungen zeigen, dass die COVID-19 Pandemie einen deutlichen Impact auf die Psychotherapiepraxis in Österreich hat. Weitere quantitative und qualitative Auswertungen der Daten werden noch tiefergehende Erkenntnisse liefern.

## Abstract (en)

The Danube University Krems, in cooperation with the Austrian Federal Association for Psychotherapy, conducted three online surveys to investigate remote psychotherapy in Austria during the COVID-19 pandemic. This article summarizes the so far published results of these surveys. A total of 1547 psychotherapists took part in the first online survey, which was conducted in the first weeks of the first COVID-19 lockdown in Austria. The results showed that psychotherapists increasingly replaced psychotherapy in personal contact with psychotherapy at a distance (telephone or internet) during the lockdown. Increased stress level and job-related worries were mainly reported by psychotherapists who were financially reliant on the income gained from psychotherapy. The experiences with psychotherapy at a distance were described as more positively than previously expected. Nonetheless, psychotherapy at a distance was viewed as not fully comparable to face-to-face psychotherapy sessions. In a second survey, which was conducted after the first lockdown ended (summer 2020), 222 Austrian psychotherapists participated. The aim of this survey was to examine the change in treatment format (personal to digital or digital to personal) more closely. At the same time as the second survey, a third online survey was carried out in which 139 patients of the 222 Austrian psychotherapists took part in order to examine the patients’ perspective on changing the treatment format. First results show that psychotherapists and patients experienced a difference in terms of the therapeutic interventions used when changing the treatment format. Moreover, the subjective meaning of different areas of life was changed in the COVID-19 pandemic compared to the time before COVID-19. The evaluations to date show that the COVID-19 pandemic has a significant impact on the psychotherapeutic practice in Austria. Further quantitative and qualitative evaluations of the data will provide even more in-depth knowledge.

## Einleitung

Die durch das Coronavirus SARS-CoV‑2 verursachte Infektionskrankheit COVID-19 hat weltweit weitreichende gesundheitliche, soziale sowie wirtschaftlichen Auswirkungen. Um die unkontrollierte Ausbreitung des Virus zu bekämpfen, wurden in vielen Ländern Maßnahmen zur Reduktion sozialer Kontakte implementiert. Aktuelle Übersichtsarbeiten und Meta-Analysen zeigen, dass die COVID-19 Pandemie und die damit einhergehenden Maßnahmen zur Eindämmung des Virus mit einem Anstieg an psychischen Problemen einhergehen (Brooks et al. 2020; Salari et al. 2020). Auch in der österreichischen Allgemeinbevölkerung konnte ein deutlicher Anstieg an Depressionen, Angststörungen und Schlafstörungen beobachtet werden (Pieh et al. 2020, 2021; Probst et al. 2020a). Diese Situation stellt Psychotherapeut*innen vor eine Herausforderung. Traditionell findet Psychotherapie in Psychotherapieräumen im direkten persönlichen Kontakt zwischen Psychotherapeut*in und Patient*in statt. Der Wechsel des Psychotherapieformats auf Telefon oder Internet ist die naheliegendste Lösung die psychotherapeutische Versorgung während einer Pandemie sicherzustellen und gleichzeitig die notwendigen Maßnahmen zur Eindämmung des Virus umzusetzen (Wind et al. 2020). Um persönliche Kontakte möglichst zu reduzieren, ist weltweit ein Trend hin zu Psychotherapie auf Distanz (insb. via Videokonferenz) zu beobachten (Humer und Probst 2020). In Österreich schließt die Internetrichtlinie des Bundesministeriums für Soziales, Gesundheit, Pflege und Konsumentenschutz (BMSGPK) Psychotherapie über das Internet jedoch aus (Bundesministerium für Gesundheit und Frauen 2005). Diese Richtlinie verlor auch während der COVID-19 Pandemie grundsätzlich nicht an Gültigkeit, vom BMSGPK kam jedoch die Ergänzung, dass in dringenden Fällen der Einsatz elektronischer Medien (Videokonferenz, Telefon) als Überbrückung in einer Notstandslage möglich ist. Zusätzlich begannen Krankenkassen für die Zeit der COVID-19 Pandemie psychotherapeutische Behandlungen via Internet oder Telefon in gleicher Form als Standard-Psychotherapien im direkten persönlichen Kontakt zu honorieren. Neben rechtlichen und finanziellen Rahmenbedingungen, bestehen auch gewisse Vorbehalte gegenüber Psychotherapie auf Distanz, da der direkte persönliche Kontakt zwischen Patient*in und Psychotherapeut*in oft als essenzieller Teil der Therapie angesehen wird (Apolinário-Hagen et al. 2018; Connolly et al. 2020; Schuster et al. 2018). Insgesamt zeigen Studien, dass Psychotherapeut*innen Psychotherapie auf Distanz kritischer gegenüberstehen als Ihre Patient*innen. Psychotherapie auf Distanz kann dabei meist gleich wirksam wie im direkten persönlichen Kontakt durchgeführte Psychotherapie sein (Connolly et al. 2020).

Um Psychotherapie in Österreich während der Pandemie zu untersuchen, wurden von der Donau-Universität Krems in Kooperation mit dem Österreichischen Bundesverband für Psychotherapie drei Onlinebefragungen zur Psychotherapie in Österreich während der COVID-19 Pandemie durchgeführt. In dieser Arbeit werden die bisherigen Ergebnisse und Publikationen dieser Befragungen zusammengefasst dargestellt.

## Methode

Die Onlinebefragungen wurden mit dem an der Donau-Universität Krems gehosteten REDCap (Harris et al. 2009, 2019) Umfragetool durchgeführt. Sowohl Datenschutzbeauftragte als auch Ethikkommission der Donau-Universität Krems haben den Onlinebefragungen zur Untersuchung der Psychotherapie in Österreich während der COVID-19 Pandemie ein positives Votum ausgestellt.

Die Details zu den Online-Umfragen sind den entsprechenden Publikationen zu entnehmen. Nachfolgend werden die zentralen Ergebnisse zusammengefasst.

### Online Umfrage 1

Eine erste Online-Umfrage mit insgesamt 79 Fragen wurde in den ersten Wochen des COVID-19 Lockdowns in Österreich (24. März 2020 bis 1. April 2020) durchgeführt. Alle Psychotherapeut*innen, die eine gültige E‑Mail-Adresse in der Psychotherapeut*innenliste des BMSGPK angegeben hatten (ca. 6000), wurden kontaktiert. Insgesamt 1547 Psychotherapeut*innen haben die Umfrage erfolgreich abgeschlossen, was einer Rücklaufquote von ca. 25 % entspricht. Die Umfrage enthielt demografische Fragen (Alter, Geschlecht, Fachspezifikum, Jahr des Eintrags in die Liste der Psychotherapeut*innen etc.).

Weitere Fragen bezogen sich auf die durchschnittliche Anzahl an Patient*innen pro Woche pro Therapieformat (persönlich, Telefon, Internet) in den Monaten vor der COVID-19 Pandemie bzw. in der aktuellen Situation um COVID-19.

Psychotherapeut*innen wurden gebeten auf einer Skala von 0 bis 100 anzugeben, wie sie über Telefon/Internet im Vergleich zu persönlichem Kontakt psychotherapeutisch behandeln können (0 = überhaupt nicht vergleichbar mit Psychotherapie in persönlichem Kontakt, 100 = voll und ganz vergleichbar zu Psychotherapie in persönlichem Kontakt). Auch ob die tatsächlichen Erfahrungen mit Psychotherapie über Telefon/Internet negativer/positiver waren als früher erwartet, wurde auf einer Skala von 0 bis 100 abgefragt (0 = deutlich negativer als erwartet, 100 = deutlich positiver als erwartet).

Weitere Fragen bezogen sich darauf, wie gut informiert sich Psychotherapeut*innen über Psychotherapie über Internet fühlten (Skala von 0 bis 100), ob zusätzlicher Informationsbedarf besteht (Skala von 0 bis 100) und welche Informationen konkret wünschenswert wären (offene Frage).

Psychotherapeut*innen sollten auch auf einer Skala von 0 bis 100 angeben, wie groß Ihre Angst ist, sich während der Psychotherapie im persönlichen Kontakt mit COVID-19 anzustecken.

Sie wurden auch gebeten, anzugeben wie gut sie die fünf von der österreichischen Regierung empfohlenen Schutzmaßnahmen gegen COVID-19 während der Psychotherapie im persönlichen Kontakt umsetzten können. Für jede der fünf empfohlenen Maßnahmen (1: regelmäßig Hände reinigen, 2: Mindestabstand von einem Meter einhalten, 3: Gesicht nicht mit Händen berühren, 4: Atemhygiene einhalten, 5: bei Symptomen zu Hause bleiben) wurde auf einer 4‑stufigen Skala abgefragt wie gut die jeweilige Maßnahme während psychotherapeutischer Behandlungen im persönlichen Kontakt umgesetzt werden kann.

Die Umfrage erhielt auch etablierte psychometrische Fragebögen zur Erfassung von Stress (PSS-10 (Cohen et al. 1983)) und berufsbezogenen Ängsten (JAS (Linden und Muschalla 2012)), welche aus jeweils 10 Items bestanden die auf einer 5‑stufigen Likert-Skala beantwortet wurden.

Diese Umfrage wurde zudem in Deutschland (19. Mai 2020 bis 28. Mai 2020), in Tschechien (6. Mai bis 20. Mai 2020) und in der Slowakei (8. Mai 2020 bis 22. Mai 2020) durchgeführt. Zum Zeitpunkt der Umfrage in diesen Nachbarländern waren die strengen Lockdown-Maßnahmen der jeweiligen Länder bereits gelockert und es bestanden keine Ausgangsbeschränkungen mehr. Insgesamt nahmen 130 Psychotherapeut*innen aus Deutschland, 112 aus Tschechien und 96 aus der Slowakei teil.

### Onlinebefragung 2

In einer weiteren österreichischen Umfrage nach dem Lockdown (26. Juni 2020 bis 3. September 2020) wurde der Wechsel des Behandlungsformats (persönlich zu digital oder digital zu persönlich) genauer untersucht. Alle Psychotherapeut*innen, die eine gültige E‑Mail-Adresse in der Psychotherapeut*innenliste des BMSGPK angegeben hatten wurden kontaktiert. An der Umfrage nahmen 222 Psychotherapeut*innen teil (ca. 4 % der kontaktierten Psychotherapeut*innen). Die Umfrage enthielt insgesamt 128 Fragen. In diesem Artikel wird nur auf die Auswertung der 30 Fragen, die sich auf die durchgeführten Interventionen beziehen, sowie auf die 12 selbstkonstruierten Fragen zur subjektiven Bedeutung verschiedener Lebensbereiche/Wertbegriffe, näher eingegangen, da Auswertungen dazu bereits publiziert sind. Die angewandten Interventionen in den Psychotherapie-Sitzungen wurden mit dem sog. MULTI-30 (Multitheoretical List of Therapeutic Interventions (Solomonov et al. 2019)) erhoben. Dieser Fragebogen besteht aus 30 Items, die 8 verschiedene Skalen ergeben (psychodynamische Interventionen, allgemeine Faktoren „common factors“, person-zentrierte Interventionen, prozess-experientielle Interventionen, Interventionen der interpersonellen Therapie, kognitive Interventionen, behaviorale Interventionen, dialektisch-behaviorale Interventionen). Psychotherapeut*innen wurde gebeten anzugeben, wie typisch die Intervention für Psychotherapie im persönlichen Kontakt bzw. auf Distanz war. Weiters sollten Psychotherapeut*innen angeben, wie wichtig verschiedene Lebensbereiche/Wertbegriffe für sie selbst sind (1: Arbeit; 2: Beziehung; 3: Bekannte, Freunde; 4: Freizeit, Hobbys; 5: Körperliche Gesundheit; 6: Psychische Gesundheit). Die Frage wurde in Bezug auf zwei unterschiedliche Zeithorizonte gestellt: vor der aktuellen Corona-Krise vs. in der aktuellen Corona-Krise.

### Onlinebefragung 3

Psychotherapeut*innen der zweiten Onlinebefragung wurden gebeten, ihre Patient*innen zu motivieren an einer dritten Onlinebefragung nach dem Lockdown (26. Juni 2020 bis 3. September 2020) teilzunehmen, um auch aus deren Perspektive den Wechsel des Behandlungsformats zu untersuchen. Insgesamt nahmen 139 Patient*innen der 222 österreichischen Psychotherapeut*innen teil. Die Umfrage bestand aus 159 Items, wobei auch hier die Patient*innen-Version des MULTI-30 (Solomonov et al. 2019), sowie die selbstkonstruierten Fragen zur subjektiven Bedeutung verschiedener Lebensbereiche/Wertbegriffe inkludiert waren.

## Ergebnisse

### Onlinebefragung 1

Die erste Onlinebefragung zeigte, dass Psychotherapeut*innen in Österreich während des Lockdowns im Vergleich zu den Monaten davor Psychotherapie im persönlichen Kontakt reduziert und Psychotherapie über Telefon und Internet erhöht haben (Probst et al. 2020b). So nahm in Österreich die Zahl der Patient*innen, die im persönlichen Kontakt therapiert wurden, in den ersten Wochen der COVID-19 Ausgangsbeschränkungen um durchschnittlich 81 % ab (*p* < 0,001), während ein deutlicher Anstieg bei Patient*innen, die über Telefon (+979 %) oder Internet (+1561 %) therapiert wurden (*p* < 0,001), verzeichnet wurde. Insgesamt konnte die Zunahme der Psychotherapie auf Distanz die Abnahme der Psychotherapie im persönlichen Kontakt nicht kompensieren, sodass in Österreich während der ersten Wochen des COVID-19 bedingten Lockdowns durchschnittlich 28 % weniger Patient*innen therapiert wurden als in den Monaten davor (*p* < 0,001). Die therapeutische Orientierung im Sinne der vier Therapie-Cluster (psychodynamisch, humanistisch, systemisch, verhaltenstherapeutisch) hatte keinen Einfluss auf die beobachteten Ergebnisse.

Die Befragung zeigte auch Unterschiede zwischen den untersuchten Ländern, sowie zwischen den Geschlechtern auf (Humer et al. 2020a). In Deutschland war mit einer durchschnittlichen Reduktion von 18 % der insgesamt geringste Rückgang an Psychotherapie im persönlichen Kontakt zu beobachten. So wurden aufgrund der Steigerung der Psychotherapien auf Distanz (v. a. über das Internet) zwei Wochen nach Ende des Lockdowns im Durschnitt um 12 % mehr Patient*innen therapiert als vor Beginn der COVID-19 Pandemie (*p* = 0,014). In Tschechien konnte keine signifikante Änderung in der Anzahl der therapierten Patient*innen beobachtet werden (*p* = 0,086), da die Reduktion der Therapien im persönlichen Kontakt (−71 %) durch die Zunahme von Therapien über Telefon und Internet kompensiert wurde. Im Gegensatz dazu, berichteten Psychotherapeut*innen aus der Slowakei drei Wochen nach dem Ende der starken Ausgangsbeschränkungen eine Abnahme der Gesamtzahl der therapierten Patient*innen von 25 % (*p* < 0,001), da die starke Abnahme der Therapien im persönlichen Kontakt (−76 %) nicht durch den Anstieg von Psychotherapien auf Distanz ausgeglichen werden konnte.

Über alle Länder hinweg wurden geschlechterspezifische Unterschiede beobachtet. So reduzierten Frauen in Zeiten von COVID-19 Psychotherapie im persönlichen Kontakt stärker (*p* = 0,036) als ihre männlichen Kollegen und stiegen verstärkt auf Psychotherapie über Telefon um (*p* = 0,015). Auch die Angst, sich während der Psychotherapie im persönlichen Kontakt mit COVID-19 anzustecken, war bei weiblichen Psychotherapeut*innen stärker ausgeprägt als bei männlichen (*p* = 0,021).

Ein weiteres wichtiges Ergebnis der ersten Befragung war, dass ein erhöhtes Stresslevel und jobbezogene Ängste v. a. bei jenen Psychotherapeut*innen in Österreich vorhanden waren, für die Psychotherapie die einzige Einnahmequelle darstellte (*p* ≤ 0,020). Stresslevel und Jobängste der Psychotherapeut*innen unterschieden sich jedoch nicht zwischen Psychotherapeut*innen, die nur im persönlichen Kontakt therapierten, die sowohl im persönlichen Kontakt als auch auf Distanz therapierten, die nur auf Distanz therapierten und jenen, die während der Ausgangsbeschränkungen keine Psychotherapien durchführten (*p* = 0,223). Konnten während der Psychotherapie im persönlichen Kontakt die von der österreichischen Regierung vorgeschlagenen Schutzmaßnahmen besser eingehalten werden, reduzierte dies die Angst sich mit COVID-19 in Psychotherapien im direkten persönlichen Kontakt mit Patient*innen zu infizieren (*p* < 0,01; Probst et al. 2020c).

Insgesamt war ersichtlich dass Psychotherapeut*innen in Österreich mit Psychotherapie über Internet positivere Erfahrungen gemacht haben als sie erwartet hätten und dass Psychotherapie über Telefon und Internet aber nicht vollständig vergleichbar ist mit Psychotherapie im persönlichen Kontakt (*p* < 0,001; Humer et al. 2020b). Es konnten auch Unterschiede zwischen den vier Therapie-Clustern festgestellt werden (*p* = 0,001). So beurteilten psychodynamisch orientierte Psychotherapeut*innen Psychotherapie via Telefon am positivsten, während Verhaltenstherapeuten dieses Format am negativsten beurteilten. Allgemein wurde Psychotherapie über Internet positiver beurteilt als Psychotherapie über Telefon (*p* < 0,001). Verhaltenstherapeut*innen (Österreich und Deutschland) erlebten Psychotherapie über Internet und Telefon umso positiver und vergleichbarer mit Psychotherapie im persönlichen Kontakt, je mehr Patient*innen sie über Internet und Telefon behandelten und – bzgl. Internet-Psychotherapie in Österreich – je besser sie sich informiert fühlten (Korecka et al. 2020).

Insgesamt gaben die Psychotherapeut*innen an sich vor allem in Bezug auf Sicherheit und Datenschutz sowie Computerprogramme mehr Information über Psychotherapie über Internet zu wünschen. Skype sowie Zoom waren die häufigsten Programme, die in Österreich für Psychotherapie über Internet verwendet wurden (Humer et al. 2020c).

### Onlinebefragung 2 und 3

Erste Ergebnissen der Onlinebefragung 2 und 3 zeigten bei der Angabe zur Wichtigkeit sechs verschiedener Bereiche des eigenen Lebens, dass Psychotherapeut*innen allgemein höhere Werte angaben als ihre Patient*innen (*p* < 0,001). Beim Vergleich der aktuellen Sichtweise im Vergleich zur Zeit vor der COVID-19 Pandemie zeigte sich, dass sowohl Psychotherapeut*innen als auch Patient*innen eine Veränderung hinsichtlich der Bedeutung diverser Lebensbereiche infolge der COVID-19 Pandemie erleben. So wurde der Bereich Arbeit von Psychotherapeut*innen und Patient*innen während COVID-19 als weniger wichtig erachtet, während psychische und physische Gesundheit an subjektiver Bedeutsamkeit gewannen (*p* < 0,001) (Humer et al. 2020d).

Die Analysen der Angaben zu den angewandten Interventionen in den Psychotherapie-Sitzungen zeigen, dass Psychotherapeut*innen und Patient*innen beim Wechsel des Behandlungsformats einen Unterschied hinsichtlich der angewandten therapeutischen Interventionen erlebten (Probst et al. 2021). So bewerteten die Psychotherapeut*innen alle untersuchten therapeutischen Interventionen als typischer für die Therapie im direkten persönlichen Kontakt als für die Psychotherapie auf Distanz (*p* < 0,001). Bei den Patient*innen waren die Unterschiede im Erleben der therapeutischen Interventionen nicht im gleichen Ausmaß sichtbar wie bei den Therapeut*innen.

## Diskussion

Die beobachteten Veränderungen im Format, in dem Psychotherapie während der ersten Monate der COVID-19 Pandemie angeboten wurde, decken sich mit anderen internationalen Studien. So wurde mit dem Ausbruch der COVID-19 Pandemie ein weltweiter Trend in Richtung Psychotherapie auf Distanz beobachtet (Wind et al. 2020). Die Ergebnisse zeigen, dass Psychotherapeut*innen sehr flexibel auf die veränderten Rahmenbedingungen reagierten und im Großen und Ganzen positiv von den neuen Behandlungsformaten überrascht waren, wenngleich Psychotherapie auf Distanz nicht komplett mit der persönlichen Psychotherapie vergleichbar angesehen wird (Humer et al. 2020b). Auch diese Befunde decken sich mit der internationalen Literatur (Connolly et al. 2020).

Insgesamt konnten Unterschiede zwischen den Ländern beobachtet werden. Während in Deutschland während der COVID-19 Pandemie sogar durchschnittlich mehr Patient*innen pro Woche therapiert wurden als in den Zeiten vor COVID-19, gab es in Tschechien keinen Unterschied, während in Österreich und der Slowakei ein Rückgang zu beobachten war. Ein Grund für die Unterschiede könnte auf den Zeitpunkt der Erhebung zurückzuführen sein. Während die Befragung in Österreich zu Beginn des ersten Lockdowns stattfand, wurden Psychotherapeut*innen in Deutschland, Tschechien und der Slowakei nach den jeweiligen ersten Lockdowns befragt. Es ist anzunehmen, dass sich Präferenzen in Bezug auf das Behandlungsformat, sowie auch Ängste vor einer COVID-19 Infektion im Zeitverlauf verändern. Weiters könnten länderspezifische gesetzliche und finanzielle Regelungen zur Psychotherapie über das Internet oder Telefon zu den Unterschieden beigetragen haben. Auch Unterschiede in Bezug auf die Angst sich während der Therapie in persönlichem Kontakt mit COVID-19 anzustecken, könnten die beobachteten Unterschieden erklären. So wurden bei deutschen Psychotherapeut*innen die geringsten Infektionsängste angegeben, was mit der Beobachtung einhergeht, dass in Deutschland die geringste Verringerung in den Psychotherapien im persönlichen Kontakt festgestellt wurde (Humer et al. 2020a). Letztlich kann auch die Nutzung digitaler Medien und somit die bereits vor der Pandemie bestehende Affinität zu digitalen Medien von Land zu Land unterschiedlich sein.

Um bestmögliche psychotherapeutische Leistungen zu gewährleisten, ist auch die Psychohygiene von Psychotherapeut*innen von großer Bedeutung. Ein Teil der Erhebung fokussierte daher auch auf Stress und berufsbezogene Ängste. Interessanterweise war das Stresserleben der Psychotherapeut*innen nicht vom Format, in welchem Psychotherapie während des Lockdowns angeboten wurde, abhängig. Es zeigte sich jedoch, dass jene Psychotherapeut*innen besonders gestresst waren und berufsbezogene Ängste hatten, deren therapeutische Tätigkeit die einzige Einnahmequelle darstellte (Probst et al. 2020c). Dieses Ergebnis verdeutlicht, dass es besonders wichtig ist finanzielle Stressoren zu reduzieren, da erhöhtes Stresserleben der Psychotherapeut*innen mit nachteiligen Auswirkungen auf den therapeutischen Prozess verbunden sein kann (Kitchingman et al. 2018; West und Shanafelt 2007).

Die Erhebungen nach Ende des ersten Lockdowns im Sommer 2020 zeigten, dass sich die therapeutischen Interventionen zwischen Behandlungsformaten (im direkten persönlichen Kontakt vs. auf Distanz) unterscheiden. So wurden die acht abgefragten therapeutischen Interventionen bei Therapie in direkter persönlicher Anwesenheit als typsicher erlebt als bei Therapie auf Distanz. Diese Unterschiede wurden vor allem aus Sicht der Therapeut*innen und weniger deutlich aus Sicht der Patient*innen angegeben (Probst et al. 2021). Mögliche Gründe könnten sein, dass die therapeutischen Interventionen nicht entsprechend aus der Ferne durchgeführt werden können bzw. Therapeut*innen in ihrer Psychotherapie-Ausbildung in Österreich nicht spezifisch auf die Anwendung im psychotherapeutischen Setting auf Distanz („remote psychotherapy“) trainiert wurden. Weiters könnten auch die Themen der Therapie und somit auch die erforderlichen Interventionen sich zwischen den Formaten unterscheiden. So wäre es z. B. denkbar, dass in Zeiten des Lockdowns, in denen auch vermehrt Psychotherapie auf Distanz angeboten wurde, vermehrt Kriseninterventionen durchgeführt wurden.

Die Erhebungen der zweiten und dritten Befragung zeigten auch auf, dass sich sowohl Psychotherapeut*innen als auch Patient*innen in sehr ähnlichen Situationen in Bezug auf die Veränderungen im subjektiven Sinn des Lebens befinden (Humer et al. 2020d). So gaben sowohl Patient*innen als auch Therapeut*innen an seit Beginn der COVID-19 Pandemie den Bereichen psychische und physische Gesundheit höhere Bedeutung zuzuordnen, während die Arbeit an subjektiver Wichtigkeit verlor.

Insgesamt müssen bei der Interpretation der Studien einige Limitationen berücksichtigt werden. So wurden alle Studien in Form von Querschnittsstudien durchgeführt und alle Daten zu Zeiten vor COVID-19 nicht direkt erfasst, sondern retrospektiv abgefragt. Weiters wurden alle Angaben auf Basis von Selbstberichten gemacht, was deren Objektivität einschränkt. Es sind keine Aussagen zur Wirksamkeit der Psychotherapien auf Basis dieser Online-Erhebungen möglich. Um Aussagen zum Prozess und Ergebnis von Psychotherapie unter Praxisbedingungen in Österreich im Längsschnitt zu erforschen, führen wir aktuell die POPP-Studie (**P**rozess und **O**utcome in **p**sychotherapeutischen **P**raxen; www.facebook.com/poppstudie) durch und laden ab sofort bis 2023 interessierte Psychotherapeut*innen ein, daran teilzunehmen. Die Online-Durchführung könnte möglicherweise auch zu einem Selektionsbias geführt haben (Bethlehem 2010). So ist es möglich, dass verstärkt Psychotherapeut*innen mit einer hohen Affinität zu digitalen Medien erreicht wurden. Dies könnte dazu beigetragen haben, dass Psychotherapie auf Distanz insgesamt eher positiv bewertet wurde. Weiters könnte es auch sein, dass aufgrund der Online-Durchführung ältere, weniger technisch affine Psychotherapeut*innen unterrepräsentiert waren. Zudem waren in den Umfragen Psychotherapeut*innen mit humanistischer Orientierung überrepräsentiert im Vergleich zur Verteilung der vier Therapie-Cluster in der österreichischen Psychotherapeut*innen-Liste. Aufgrund dieser Einschränkungen ist es möglich, dass die Umfragen kein repräsentatives Bild der Psychotherapie in Österreich während COVID-19 darstellen.

## Schlussfolgerung

Die bisherigen Auswertungen zeigen, dass COVID-19 einen deutlichen Impact auf die Psychotherapiepraxis in Österreich hat. So wurde während des ersten COVID-19 bedingten Lockdowns Psychotherapie verstärkt über Internet und Telefon angeboten. Die Erfahrungen der Psychotherapeut*innen mit Therapie auf Distanz sind über alle therapeutischen Orientierungen hinweg sehr positiv, wenngleich Psychotherapie im persönlichen Kontakt als ideale Therapieform angesehen wird. Weitere quantitative und qualitative Auswertungen der Daten werden noch tiefergehende Erkenntnisse liefern.
